# Omission of contralateral biopsies in unilateral MRI‐suspicious prostate cancer has minimal impact on clinical risk assessment

**DOI:** 10.1002/bco2.70154

**Published:** 2026-02-04

**Authors:** Stijn M. van den Bosch, Daniël L. van den Kroonenberg, Bram W. van Bruggen, Katelijne C. C. de Bie, Auke Jager, Arnoud W. Postema, Daniela E. Oprea‐Lager, Ivo G. Schoots, Jorg R. Oddens, Andre N. Vis

**Affiliations:** ^1^ Amsterdam UMC, Location VUmc Amsterdam The Netherlands; ^2^ Cancer Centre Amsterdam Amsterdam The Netherlands; ^3^ Leiden University Medical Center Department of Urology Leiden The Netherlands; ^4^ Department of Radiology and Nuclear Medicine Radboud UMC Nijmegen The Netherlands; ^5^ Department of Radiology and Nuclear Medicine Erasmus MC Rotterdam The Netherlands; ^6^ Prostate Cancer Network Netherlands Amsterdam The Netherlands

**Keywords:** MRI, perilesional biopsy, prostate biopsy, prostate cancer, systematic biopsy, targeted biopsy, unilateral lesion

## Abstract

**Background and Objective:**

MRI‐targeted biopsies (TBx) combined with systematic biopsies (SBx) have traditionally been recommended for patients with unilateral MRI‐suspicious prostate cancer (PCa) lesions. Recent European Association of Urology (EAU) guidelines propose that, in cases with a solitary lesion, TBx with perilesional sampling may suffice, potentially omitting contralateral SBx. The clinical impact of this omission remains uncertain. This study evaluates how omitting contralateral SBx affects pathological grading, EAU risk classification, and estimated lymph node involvement (LNI).

**Methods:**

We conducted a retrospective cohort study (2016–2024) including 190 biopsy‐naïve men diagnosed with PCa via TBx and bilateral SBx for a single, unilateral MRI‐visible lesion. Outcomes were re‐evaluated using only TBx and ipsilateral SBx, simulating omission of contralateral SBx. Changes in ISUP grade, EAU risk group, and LNI probability using the Briganti 2019, Amsterdam–Brisbane–Sydney (ABS), and Memorial Sloan Kettering Cancer Center (MSKCC) nomograms were assessed. Furthermore, we compared cancer detection rates for TBx alone versus TBx plus ipsilateral SBx.

**Key Findings:**

ISUP grading changed in 14 of 190 patients (7.4%) when contralateral SBx was omitted; two patients (1.1%) had clinically significant PCa (ISUP 2) detected solely in contralateral cores. In seven patients (3.7%), only ISUP 1 cancer was found in contralateral SBx, meaning no diagnosis would have been made without those cores. EAU risk classification changed in five patients (2.6%). LNI estimates were affected minimally: One patient (0.5%) dropped below the Briganti 7% threshold, and one patient (1.2%) no longer met the 8% ABS threshold. The MSKCC model showed a statistically significant 8% increase in patients above the 7% ePLND threshold when contralateral SBx was omitted (*p* = 0.0023). Ipsilateral SBx substantially improved detection of higher‐grade disease: sensitivity for ISUP ≥3 increased from 82% with TBx alone to 98% when combined with ipsilateral SBx.

**Conclusion and Clinical Implications:**

Omitting contralateral SBx in patients with unilateral MRI lesions had limited effect on grading, risk classification, and LNI estimates. However, ipsilateral SBx adds significant diagnostic value compared with TBx alone and should be retained. These findings support a more targeted diagnostic approach in patients with a unilateral lesion on MRI in a primary diagnostic setting.

## 1 INTRODUCTION

Prostate cancer (PCa) is among the most prevalent malignancies affecting men globally, with an estimated 1.4 million new cases and 375 000 deaths worldwide in 2020.^1^ The European Association of Urology (EAU) Guidelines on PCa recommends using magnetic resonance imaging (MRI) in case of elevated prostate‐specific antigen (PSA) or suspicious digital rectal examination (DRE). Traditionally, PCa diagnosis has relied on a combination of MRI‐targeted prostate biopsies (TBx) and bilateral systematic biopsies (SBx). However, the necessity and clinical impact of performing contralateral SBx in patients with a unilateral (visible) lesion on MRI remains subject to ongoing debate.^2^ A recent meta‐analysis showed no decrease in detection rate of clinically significant PCa (csPCa) when omitting contralateral SBx compared with bilateral SBx with TBx.^3^ Following this study, EAU guidelines now recommend TBx with perilesional (or ipsilateral SBx) approach in patients with elevated PSA levels and unilateral suspicious findings on MRI (i.e., a PIRADS 4/5) lesion.^1^

This new approach could reduce the overdiagnosis of clinically insignificant prostate cancer, which is often associated with SBx.^4^ However, the rationale for performing contralateral SBx could be that the diagnostic information gained by additional biopsies of the contralateral side may give a more proper identification of tumour grade and risk classification which could guide treatment planning, potentially impacting eligibility for active surveillance, planning of surgical intervention, or radiation therapy.

However, recent studies suggest that omitting contralateral SBx in patients with unilateral MRI‐visible lesions has only limited impact on overall treatment planning.^5^, ^6^ Most of these studies have focused on decisions such as active surveillance or definitive treatment, while giving less attention to the implications for lymph node dissection. Yet, biopsy results are also essential for estimating the probability of lymph node involvement (LNI), which directly influences the indication for extended pelvic lymph node dissection (ePLND).^7^, ^8^, ^9^ In particular, the number and proportion of positive biopsy cores are key inputs in several LNI prediction nomograms. To date, the specific impact of omitting contralateral SBx on these diagnostic models, and thereby on surgical planning, remains unclear.

This study addresses the impact of omitting contralateral SBx in patients with unilateral suspicious MRI lesions on PCa grading, risk group stratification, and estimated LNI probability.

## 2 METHODS

### 2.1 Study population and design

This retrospective cohort study was conducted at a tertiary academic hospital in the Netherlands and included patients with suspected PCa who underwent both MRI‐TBx and bilateral SBx between 2016 and 2024. Eligible patients had elevated PSA levels and a single, unilateral, MRI‐visible lesion classified as PI‐RADS 3–5. All were biopsy‐naïve at the time of inclusion.

Patients were excluded if they had undergone prior PCa treatment, had lesions crossing the prostate midline, or had multiple suspicious lesions. Of more than 1500 patients biopsied during the study period, 190 met these criteria. Lesion multiplicity and laterality were independently assessed by two uroradiologists using PI‐RADS v2.1. Approximately 40% of MRIs were acquired using 1.5‐T scanners without dynamic contrast enhancement, which may have affected lesion conspicuity and is acknowledged as a study limitation.

To assess the clinical impact of omitting contralateral SBx, each patient served as their own control. Outcomes were compared between two scenarios: (1) full biopsy data (TBx + bilateral SBx) and (2) a hypothetical scenario excluding contralateral SBx, using only TBx and ipsilateral SBx results. This within‐patient design allowed for direct evaluation of changes in ISUP grade, EAU risk classification, and LNI probability based on established nomograms.

### 2.2 MRI protocol

The acquisition of MRI images was conducted in accordance with the PI‐RADS V2.1 guidelines.^10^ The MRI sequences employed included T1‐weighted, T2‐weighted, diffusion‐weighted imaging, and apparent diffusion coefficient maps (1.5 or 3 Tesla).

### 2.3 Biopsy protocol and histopathological assessment

Between 2016 and 2024, prostate biopsies were performed using a transrectal or transperineal approach, depending on the time period. Until July 2020, transrectal prostate biopsies were routinely performed. Thereafter, the institution transitioned to the transperineal technique. While both approaches employed MRI/ultrasound fusion targeting, spatial sampling accuracy and complication profiles differ.

The transrectal biopsy procedure was conducted with the patient in the left lateral decubitus position under local periprostatic nerve block anaesthesia. Imaging was performed using the Philips iU22 ultrasound system (Philips Healthcare, Best, The Netherlands), equipped with an end‐firing probe. Each procedure typically included 12 SBx cores and 2–3 TBx cores per MRI‐visible lesion. MRI‐targeting was facilitated by the Eigen Artemis® MRI/ultrasound fusion system (Eigen, Grass Valley, CA, USA) in combination with the ProFuse® software suite (Eigen, Grass Valley, CA, USA).

The transperineal biopsies were performed with the patient in the supine position, using local infiltration anaesthesia. A BK5000 ultrasound system (BK Medical, Herlev, Denmark) with a biplane transrectal probe was used, stabilized on a dedicated platform. Biopsies were taken using a brachytherapy template grid, with 14 systematic cores and two to three MRI‐targeted cores per lesion. Fusion guidance was provided using MIM Symphony Dx® software (MIM Software Inc., Cleveland, OH, USA).

All biopsy specimens were reviewed by an experienced uropathologist according to the 2019 consensus grading guidelines of the International Society of Urological Pathology (ISUP).^11^

### 2.4 Risk group classification and lymph node involvement probability

Patients were stratified into low‐, intermediate‐, or high‐risk groups based on the EAU guidelines.^1^ To estimate the probability of LNI, three externally validated nomograms were used: the Memorial Sloan Kettering Cancer Center (MSKCC) 2023, Briganti 2019, and Amsterdam–Brisbane–Sydney (ABS) models.^7^, ^8^, ^9^ These models incorporate different clinical and pathological parameters to determine the likelihood of LNI and guide decisions on the indication for ePLND in prostate cancer management.

An overview of the input parameters required for each nomogram is provided in Table 1 to illustrate how contralateral SBx results could influence their output. Since all three models partially depend on the percentage and distribution of positive biopsy cores, omitting contralateral SBx may affect the calculated probability of LNI.

**TABLE 1:** Overview of input parameters used in LNI nomograms.

Nomogram	Input parameters	ePLND threshold
MSKCC 2023	PSA level Clinical T stage ISUP grade group Percentage of positive biopsy cores Number of positive and total cores	≥7%
Briganti 2019	PSA level Clinical T stage ISUP grade group Percentage of positive biopsy cores MRI‐visible lesion presence	≥7%
ABS	PSA level Clinical T stage ISUP grade group MRI‐visible lesion presence PSMA PET findings	≥8%

In this study, each nomogram was applied to the same cohort twice: first using the complete biopsy dataset (TBx + bilateral SBx), and again using only TBx and ipsilateral SBx data. This within‐patient comparison enabled quantification of the potential impact of omitting contralateral biopsy cores on LNI estimation.

As part of institutional staging protocols, PSMA PET/CT was performed in patients with ISUP grade ≥2 disease or clinical T stage ≥2.

### 2.5 Statistical analysis

Descriptive statistics were used to summarize baseline characteristics and biopsy outcomes. To evaluate the impact of omitting contralateral SBx on the detection of clinically insignificant prostate cancer (ISUP 1), a paired McNemar's test was applied. This test compared the presence or absence of ISUP 1 detected in the full biopsy protocol (TBx + bilateral SBx) versus the limited protocol (TBx + ipsilateral SBx). A *p* value <0.05 was considered statistically significant. All statistical analyses were performed using STATA version 17 (StataCorp LLC, College Station, TX, USA).

### 2.6 Ethical approval

This retrospective study was approved by the institutional review board of Amsterdam UMC. The requirement for informed consent was waived due to the retrospective nature of the study.

## 3 RESULTS

### 3.1 Patient characteristics

Table 2 presents the baseline characteristics of 190 patients who underwent bilateral SBx + MRI‐directed TBx. The median age at biopsy was 69 years (IQR 62–74). The median prebiopsy PSA level was 8.0 μg/L (IQR 5.3–11). Digital rectal examination findings were benign in 46% (*n* = 87), suspicious in 49% (*n* = 94), and missing in 4% (*n* = 8) of patients. The median MRI prostate volume was 46 mL (IQR 32–69). The median number of systematic biopsies was 12 (IQR 12–14) and 3 (IQR 3–3) for TBx. The highest ISUP grade in all SBx and TBx was ISUP 1 in 17% (*n* = 32), ISUP 2 in 35% (*n* = 66), ISUP 3 in 24% (*n* = 46), and ISUP 4–5 in 24% (*n* = 46).

**TABLE 2:** Baseline characteristics of all patients.

	All patients (*N* = 190)
Age at biopsy—year median (IQR)	69 (62–74)
Prebiopsy PSA—μg/L median (IQR)	8.0 (5.3–10.8)
Clinical T stage—no. (%)	
cT1c	87 (46%)
cT2	85 (45%)
cT3/4	9 (4.7%)
Missing	8 (4.2%)
Biopsies MRI TBx—no. median (IQR)	3 (3–3)
Biopsies SBx—no. median (IQR)	12 (12–14)
MRI prostate volume—mL (IQR)	46 (32–69)
MRI results—no. (%)	
PIRADS 3	27 (14%)
PIRADS 4	78 (41%)
PIRADS 5	85 (45%)
Highest pathology in SBx + TBx—no. (%)	
ISUP 1	32 (17%)
ISUP 2	66 (35%)
ISUP 3	46 (24%)
ISUP 4	23 (12%)
ISUP 5	23 (12%)

Abbreviations: IQR, interquartile range; ISUP, International Society of Urological Pathology; MRI, magnetic resonance imaging; PCa, prostate cancer; PSA, prostate‐specific antigen; SBx, systematic biopsies; SD, standard deviation; TBx, targeted biopsies.

### 3.2 ISUP and EAU risk classification

Omitting contralateral SBx resulted in a change in ISUP grade in 14 of 190 patients (7.4%) (Table 3). Among these, seven patients (3.7%) had no cancer detected in the ipsilateral SBx or TBx, but ISUP 1 cancer was identified in the contralateral SBx. Additionally, ISUP grade was downgraded in seven patients when contralateral SBx data were excluded: two patients (1.1%) from ISUP 2 to ISUP 1 or no cancer, two (1.1%) from ISUP 3 to ISUP 2, two (1.1%) from ISUP 4 to ISUP 3, and one (0.5%) from ISUP 5 to ISUP 3. The number of patients classified as ISUP 1 decreased from 32 to 26 when contralateral SBx was omitted.

**TABLE 3:** Patient‐level comparison of highest prostate cancer grade groups detected by MRI‐targeted biopsy and only ipsilateral systematic biopsy versus MRI‐targeted biopsy and bilateral systematic biopsy.

		MRI‐TBx + bilateral SBx						Total
		No PCa	ISUP 1	ISUP 2	ISUP 3	ISUP 4	ISUP 5	
MRI‐TBx + ipsilateral SBx	No PCa	0	7	1	0	0	0	8
	ISUP 1		25	1	0	0	0	26
	ISUP 2			64	2	0	0	66
	ISUP 3				44	2	1	47
	ISUP 4					21	0	21
	ISUP 5						22	22
Total		0	32	66	46	23	23	190

Abbreviations: ISUP, International Society Urology Pathology; PCa, prostate cancer; SBx, systematic biopsies; TBx, target biopsies.

Regarding EAU risk classification, 17% of patients (*n* = 32) were classified as low risk, 59% (*n* = 112) as intermediate risk, and 24% (*n* = 46) as high risk based on bilateral SBx and TBx data (Table 4). Omitting contralateral SBx led to EAU risk group downgrading in five patients (2.6%): two shifted from intermediate to low risk, and three from high to intermediate risk.

**TABLE 4:** Patient‐level comparison of EAU risk group detected by MRI‐targeted biopsy and only ipsilateral systematic biopsy versus MRI‐targeted biopsy and bilateral systematic biopsy.

		MRI‐TBx + bilateral SBx			Total
		Low risk	Intermediate risk	High risk	
MRI‐TBx + ipsilateral SBx	Low risk	32	2	0	34
	Intermediate risk	0	110	3	113
	High risk	0	0	43	43
Total		32	112	46	190

Abbreviations: EAU, European Association of Urology; MRI, magnetic resonance imaging; SBx, systematic biopsies; TBx, targeted biopsies.

### 3.3 Additional value of ipsilateral systematic biopsy

Detection rates were compared between TBx alone, TBx combined with ipsilateral SBx, and TBx combined with bilateral SBx (Table 5). The combination of TBx with bilateral SBx served as the reference standard. For ISUP grade group 1 (*n* = 32), the sensitivity of TBx alone was 75% (95% CI 58–88), which increased to 84% (95% CI 67–95) when combined with ipsilateral SBx. For ISUP grade group ≥2 (*n* = 158), TBx alone detected 92% (95% CI 87–96) of clinically significant prostate cancers, improving to 99% (95% CI 95–100) with the addition of ipsilateral SBx. For ISUP grade group ≥3 (*n* = 92), TBx achieved a sensitivity of 82% (95% CI 72–89), which increased to 98% (95% CI 92–100) when ipsilateral SBx was included.

**TABLE 5:** An overview of patients with a reduced lymph node involvement probability in the MSCKK, Briganti 2019, and ABS nomogram after omission of contralateral SBx.

Nomogram	MSCKK 2023		*p* value	Briganti 2019		*p* value	ABS		*p* value
Biopsy approach	Bilateral SBx + TBx	Ipsilateral SBx + TBx		Bilateral SBx + TBx	Ipsilateral SBx + TBx		Bilateral SBx + TBx	Ipsilateral SBx + TBx	
LNI probability, median (IQR)	12% (3.9–45.4)	15.9% (6.1–51.3)	<0.001	5.5% (2.3–22)	5.9% (2.5–26)	<0.001	12.5% (8.3–31.6)	14.5% (8.8–37.7)	<0.001
Threshold	7%			7%			8%		
Patient no above threshold, *n* (%)	117/190 (62%)	132/190 (70%)	0.0023	88/190 (46%)	89/190 (47%)	1.0	75/84 (89%)	74/84 (88%)	1.0

Abbreviations: ABS, Amsterdam–Brisbane–Sydney; MSCKK, Memorial Sloan Kettering Cancer Center; SBx, systematic biopsies; TBx, targeted biopsies.

### 3.4 Lymph node involvement probability

When applying the Briganti 2019 nomogram, omitting contralateral SBx results led to a change in ePLND indication in three out of 190 patients (1.6%). Specifically, two patients (1.1%) shifted from below to above the 7% threshold used to recommend ePLND, while one patient (0.5%) moved from above to below this threshold (Table 5). Although the total number of patients exceeding the threshold was nearly unchanged (88 with bilateral SBx vs. 89 with ipsilateral SBx only), this analysis highlights that treatment recommendations would have differed in three individual cases.

A PSMA PET/CT scan was performed in 84 of the 190 patients (44%), allowing for LNI probability estimation using the ABS nomogram in this subgroup. When contralateral SBx results were omitted, the ePLND indication threshold (8%) was crossed in three patients: two patients (2.4%) shifted from below to above the threshold, and one patient (1.2%) shifted from above to below. This means that in three of 84 patients (3.6%), the treatment recommendation based on LNI probability would have changed.

Using the MSKCC nomogram, omission of contralateral SBx data altered the ePLND recommendation in 21 of 190 patients (11%). Specifically, 18 patients (9.5%) dropped below the 7% threshold, thereby losing the indication for ePLND, while three patients (1.6%) rose above the threshold, gaining an indication. These shifts directly reflect changes in treatment recommendations due to the absence of contralateral biopsy information. Overall, the proportion of patients exceeding the 7% ePLND threshold increased significantly when contralateral SBx data were omitted (from 62% to 70%, *p* = 0.0023), as shown in Table 5.

## 4 DISCUSSION

This study evaluated the clinical impact of omitting contralateral SBx in biopsy‐naïve patients with a solitary, MRI‐visible prostate lesion. Our findings demonstrate that omitting contralateral SBx had a limited effect on ISUP grading, EAU risk classification, and estimated LNI probability. These results support the growing trend towards more focused, MRI‐guided biopsy strategies aimed at reducing unnecessary sampling while maintaining diagnostic accuracy.

Contralateral SBx altered ISUP grading in 14 of 190 patients (7.4%). Only three patients (1.6%) had clinically significant prostate cancer (csPCa; ISUP 2) detected exclusively in contralateral SBx cores, all based on a single positive core among two to three contralateral samples. If contralateral SBx had been omitted, these patients would have been classified as low risk and likely considered for active surveillance. However, two of these patients ultimately received curative‐intent treatment. This suggests that omission of contralateral SBx may have altered treatment decisions in up to 1.1% of patients, highlighting a rare but potentially meaningful impact. Additionally, seven patients (3.7%) were diagnosed with ISUP 1 disease exclusively through contralateral SBx. Such low‐grade, MRI‐invisible cancers are generally not considered clinically significant and are managed with active surveillance according to current guidelines. Detecting these lesions may increase the risk of overdiagnosis, leading to patient anxiety, unnecessary interventions, and potential overtreatment. Although the addition of around six contralateral cores is not expected to significantly increase complication rates, it contributes to greater patient discomfort, longer procedure times, higher pathology processing costs, and a higher risk of detecting clinically insignificant prostate cancer.

Omission of contralateral SBx led to EAU risk group reclassification in five patients (2.6%): three shifted from intermediate to low risk, and two from high to intermediate risk. In practice, this could impact both surveillance eligibility and treatment intensity. Notably, the two patients downgraded from high to intermediate risk had solitary ISUP 3 cores in the contralateral lobe. According to current EAU and NCCN guidelines, the duration of androgen deprivation therapy (ADT) given with radiotherapy depends on risk group. High risk patients receive treatment for 24 to 36 months, while intermediate risk patients may be treated for only 4 to 6 months. Therefore, omission of contralateral SBx in these cases may have led to shorter ADT courses and less intensive treatment. Additionally, contralateral TBx could potentially impact decisions on pelvic nodal radiation when LNI increases based on these biopsies. However, previous studies showed that the implications of contralateral SBx for radiotherapy planning are limited.^5^

We also examined the role of ipsilateral SBx compared with TBx alone with bilateral SBx and TBx as reference. While MRI‐TBx alone detected 92% of ISUP ≥2, combining TBx with ipsilateral SBx increased the sensitivity for detecting ISUP ≥3 from 82% to 98%. This significant improvement supports incorporating ipsilateral SBx, as these samples likely compensate for cancers missed due to targeting inaccuracies during TBx. In contrast, contralateral SBx added little to sensitivity for csPCa, underscoring the limited value when TBx and ipsilateral SBx are performed.

Although contralateral SBx may occasionally identify additional csPCa, the overall likelihood of significantly altering treatment decisions is low when comprehensive ipsilateral sampling is performed in the context of a solitary MRI‐visible lesion. Moreover, the long‐term significance of MRI‐invisible, low‐volume ISUP 2 lesions remains uncertain, particularly with respect to oncologic outcomes such as biochemical recurrence or metastasis‐free survival. While the reclassification rate was very low, further prospective follow‐up is needed to confirm the long‐term safety of omitting contralateral sampling.

Regarding LNI estimation, omission of contralateral SBx resulted in minimal changes across three established nomograms (Briganti 2019, ABS, and MSKCC). Using the Briganti nomogram, only four patients (2.1%) crossed the 7% threshold for ePLND indication; no such threshold crossings occurred using the ABS model. The MSKCC nomogram, more sensitive to the proportion of positive cores, showed the largest impact with 21 patients (11%) changing ePLND indication status. The divergence among models reflects their distinct design contexts: while the Briganti and MSKCC models were developed for surgical candidates and calibrated to detect node‐positive disease, the ABS model integrates PSMA PET/CT and uses a more conservative 8% threshold suitable for imaging‐based staging. Although these nomograms are widely used to guide ePLND decisions, it is important to note that most patients in this study did not undergo surgical nodal dissection. Thus, LNI estimates remain theoretical, and the lack of pN confirmation is a key limitation. These results should be interpreted with caution.

Our findings are consistent with recent literature advocating MRI‐targeted biopsy strategies. Studies have demonstrated that targeted biopsies with perilesional or regional sampling provide high csPCa detection rates with minimal added value from systematic contralateral sampling.^12^ In our cohort, omitting contralateral SBx reduced the number of detected ISUP 1 cases (26/190 vs. 32/190), suggesting a potential reduction in overdiagnosis and overtreatment.

However, some subgroups may still benefit from contralateral SBx. Patients with discordant clinical findings, such as elevated PSA levels despite low suspicion on MRI, or those with a high likelihood of multifocal disease may be at risk of missed csPCa. For instance, van Riel, Jager and Meijer et al. found that 17.2% of patients with negative MRI (PI‐RADS 1–2) and elevated PSA harboured csPCa on systematic biopsy.^13^ Similarly, in the context of active surveillance, contralateral sampling could identify otherwise undetected higher‐grade lesions. Yet the relevance of such findings remains unclear, and individualized decision‐making is warranted.

Several study limitations must be acknowledged. First, the retrospective design introduces a risk of selection bias, and patients were selected based on the availability of complete biopsy data. Second, biopsy protocols varied over the 8‐year study period, including a transition from transrectal to transperineal approaches, potentially affecting sampling consistency. Imaging protocols also evolved; approximately 40% of MRIs were performed using 1.5‐T scanners without dynamic contrast, possibly reducing lesion visibility and underestimating multiplicity. Although all scans were reviewed by experienced uroradiologists using PI‐RADS v2.1 criteria, subtle midline or bilateral lesions may have gone undetected.

Finally, the lack of long‐term follow‐up data on oncologic outcomes, such as recurrence and survival, limits definitive conclusions. Future prospective studies using standardized MRI and biopsy protocols are needed to validate these findings and to identify which patients may benefit from tailored biopsy strategies that minimize unnecessary procedures without compromising diagnostic accuracy.

## 5 CONCLUSION

Our study demonstrates that omitting contralateral SBx data in patients with single unilateral MRI‐detected PCa has minimal impact on ISUP grading, EAU risk classification, and estimated LNI probability. Analysis demonstrated that ipsilateral SBx significantly improved detection of csPCa compared with TBx alone. These findings support that contralateral SBx can be safely omitted in patients with a single unilateral MRI‐visible lesion in a primary diagnostic setting. This approach allows for a more efficient and focused diagnostic pathway, reducing the detection of clinically insignificant prostate cancer without compromising treatment decision‐making.

## AUTHOR CONTRIBUTIONS

**Stijn M. van den Bosch:** Conceptualisation; methodology; formal analysis; data curation; writing—original draft; writing—review and editing. **Daniël L. van den Kroonenberg:** Conceptualisation; methodology; formal analysis; data curation; writing—original draft; writing—review and editing. **Bram W. van Bruggen:** Data curation. **Katelijne C. C. de Bie:** Data curation. **Auke Jager:** Data curation; supervision. **Arnoud W. Postema:** Supervision. **Daniela E. Oprea‐Lager:** Supervision. **Ivo G. Schoots:** Supervision. **Jorg R. Oddens:** Supervision. **Andre N. Vis:** Supervision.

## CONFLICT OF INTEREST STATEMENT

The authors declare no conflicts of interest.
